# Allogenic umbilical cord tissue for temporomandibular joint injuries

**DOI:** 10.3389/fpain.2023.1281277

**Published:** 2023-10-24

**Authors:** Adarsh Aratikatla, Samir Ghandour, Nicola Maffulli, Manu Gupta, Ashim Gupta

**Affiliations:** ^1^ The Royal College of Surgeons in Ireland, Dublin, Ireland; ^2^ Faculty of Medicine, The University of Balamand, Beirut, Lebanon; ^3^ Department of Musculoskeletal Disorders, School of Medicine and Surgery, University of Salerno, Fisciano, Italy; ^4^ San Giovanni di Dio e Ruggi D’Aragona Hospital “Clinica Orthopedica” Department, Hospital of Salerno, Salerno, Italy; ^5^ Barts and The London School of Medicine and Dentistry, Centre for Sports and Exercise Medicine, Queen Mary University of London, London, United Kingdom; ^6^ School of Pharmacy and Bioengineering, Keele University School of Medicine, Stoke on Trent, United Kingdom; ^7^ Polar Aesthetics Dental & Cosmetic Centre, Noida, India; ^8^ Regenerative Orthopaedics, Noida, India; ^9^ Future Biologics, Lawrenceville, GA, United States; ^10^ BioIntegrate, Lawrenceville, GA, United States; ^11^ South Texas Orthopaedic Research Institute (STORI Inc.), Laredo, TX, United States

**Keywords:** umbilical cord, Wharton’s jelly, mesenchymal stem cells, regenerative medicine, temporomandibular joint, TMJ

## Abstract

The temporomandibular joint (TMJ) is crucial for functions of daily living such as mastication and articulation. Common TMJ issues include osteoarthritis, internal derangement, and myofascial pain dysfunction. Conservative methods such as physical therapy and medications are used, with surgical options such as arthroscopy and replacement for severe cases. Emerging regenerative medicine explores non-surgical treatments using human stem cells from umbilical cord derivatives, showing potential for tissue regeneration in TMJ disorders. A systematic search was conducted across PubMed, Embase, Scopus, and Web of Science databases, adhering to PRISMA guidelines, aiming to identify relevant articles published in English until August 2023. The search used specific terms to target *in vitro*, preclinical, and clinical studies on umbilical cord (UC)-derived tissue and mesenchymal stem cells (MSCs) for treating TMJ disorders. The search was extended to three clinical trial registries for on-going investigations related to UC tissue and MSCs for TMJ disorder management. The studies included in this article report the safety and efficacy profiles of allogenically acquired, umbilical cord-derived tissues and associated mesenchymal stem cells for temporomandibular joint ailments, future adequately powered, randomized controlled trials are warranted to conclusively justify the clinical use of this biologic therapy.

## 1. Introduction

The TMJ is a complex joint that connects the mandible to the temporal bone of the skull. It is responsible for the movement of the jaw, allowing for simple but crucial functions such as chewing, speaking, and yawning. The TMJ consists of several anatomical components, including the condyle of the mandible, the articular eminence of the temporal bone, and the articular disc that separates the two surfaces. Several common pathologies are associated with the TMJ, including osteoarthritis, internal derangement, and myofascial pain dysfunction syndrome (1–4). These pathologies can result in pain, limited jaw movement, and difficulty in performing daily activities. Commonly injured TMJ components include the articular disc, the condyle, and the surrounding ligaments and muscles (1). The causes of TMJ pathologies can vary, including trauma, degenerative changes, and inflammatory conditions (1, 5). Trauma to the TMJ, such as direct impact or dislocation, can lead to structural damage and subsequent pathologies. Degenerative changes, such as those seen in osteoarthritis, can result from wear and tear of the joint. Inflammatory conditions, such as rheumatoid arthritis, can also affect the TMJ and induce secondary pathologies of the joint. Management modalities for TMJ disorders can be categorized into conservative management and surgical interventions. Conservative management options include physical therapy, pain medications including non-steroidal anti-inflammatory drugs, and the use of oral appliances to improve jaw alignment (3). If conservative management proves inadequate or severe symptoms are present, surgical interventions may be necessary, including arthroscopy, arthroplasty, and joint replacement (6). These surgical procedures aim to repair or replace injured structural components of the TMJ to restore function and alleviate associated symptoms. Overall, the current treatment methods for TMJ disorders are limited. Conservative management may provide temporary relief but may not address the underlying cause of the pathology. Surgical interventions carry potential risks and may not always result in successful outcomes (5). Additionally, the availability and accessibility of surgical interventions may be limited in specific regions or for certain patient populations.

Regenerative medicine (RM) is an emerging field focusing on conservative, non-operative treatment for various disorders, including TMJ disorders. RM treatments involve biologics derived from perinatal tissues, including umbilical cord (UC) (7). UC derivatives, including UC blood and Wharton's jelly, contain various types of stem cells, including MSCs (7). These MSCs can differentiate into various cell types, including bone, cartilage, and muscle cells, with promising potential for tissue regeneration (8). *In vitro*, UC-derived MSCs can enhance the recovery of injured tissues, including liver and different joint tissues (9). This discovery prompted the investigation of various therapeutic applications of biologics, including UC components. The primary objective of this mini-review is to explore the existing *in vitro*, pre-clinical, and clinical literature covering the therapeutic applications of UC components for TMJ disorders. The secondary objective of this study is to list the ongoing clinical trials registered on United States and international trial protocol repositories.

## 2. Methods

### 2.1. Search criteria

**Figure 1:**
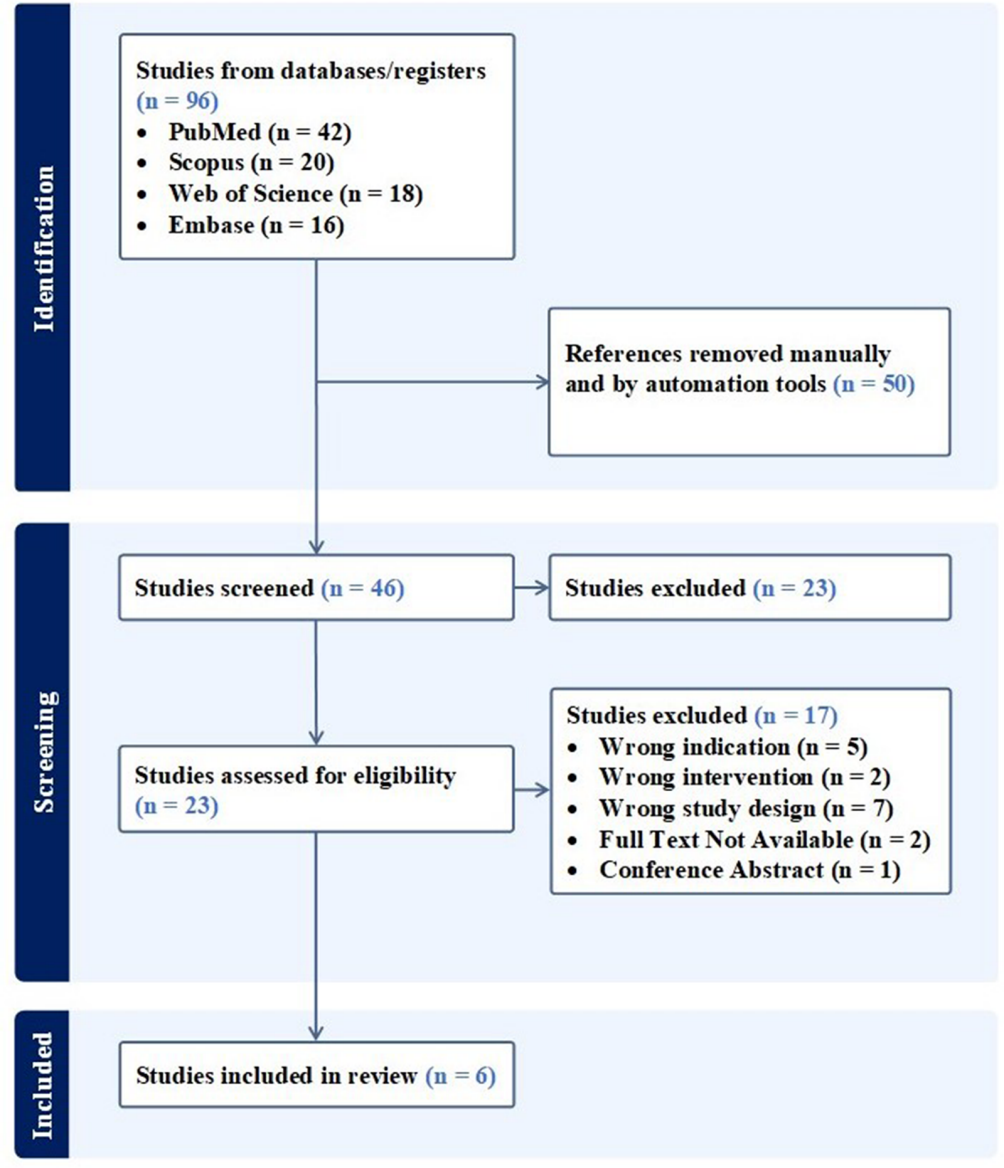
PRISMA flow diagram.

A systematic search including databases such as PubMed (MEDLINE), Embase, Scopus, and Web of Science was conducted, aiming to retrieve relevant articles published in English to August, 2023. Adherence to the PRISMA statement and guidelines was thoroughly maintained throughout the study, using the designated search terms: (“umbilical” OR “umbilical cord” OR “Wharton's jelly” OR “umbilical cord blood”) AND (“TMJ” OR “temporomandibular joint”). Eligibility criteria included studies investigating both acute and temporomandibular joint injuries, which encompass the bones (i.e., mandibular condyles, articular surface of the temporal bone), articular accessories (i.e., joint capsule, articular disc), ligaments (i.e., temporomandibular, stylomandibular, and sphenomandibular), and muscles (i.e., masseter, temporal, and medial/lateral pterygoids). The inclusion of animal and human models broadened our research scope by providing researchers’ insight into the biologic's effect in a controlled environment. Moreover, publications were required to include the use of tissue or mesenchymal stem cells (MSCs) sourced exclusively from the umbilical cord as the index intervention. Placebo, injury models including controls, acute, or chronic, and gold-standard treatment models were compared. The study selection process was carried out by two independent reviewers, utilizing dedicated reference management software to ensure the inclusion of all possible articles that fit our criteria. Figure 1 illustrates the performed search for articles. Following the specified search strategy across the four listed databases, a screening process was applied to the papers based on their titles and abstracts. Articles that did not meet our inclusion criteria were subsequently excluded. Subsequently, full-text reviews were conducted on the remaining papers to ensure alignment with the scope of our study. To address the secondary objective of this study, clinicaltrials.gov, the Chinese Clinical Trial Register (ChiCTR) and the Clinical Trials Registry—India (CTRI) were searched using the same terms to identify registered trials on the use of UC tissue and/or associated MSCs for the management of TMJ disorders.

## 3. Results

### 3.1. *In vitro* studies

Bailey et al.'s comparative study assessed whether a polyglycolic acid (PGA) scaffold seeded with human umbilical cord matrix (hUCM) stem cells (*n* = 3.4 × 10^6^ cells) or TMJ condylar chondrocytes (*n* = 3.4 × 10^6^ cells) produced better results for TMJ cartilage regeneration. hUCM-MSCs were harvested from umbilical cords obtained from a local obstetrician by washing, disinfecting, enzymatically digesting, mincing, and plating the tissue on a suitable medium. The processed MSCs were then maintained and passaged in the enriched media. To obtain TMJ chondrocytes, porcine TMJ cartilage was harvested, enzymatically digested, cultured, passaged, and eventually plated in a medium enriched with transforming growth factor beta-1 and insulin transferrin-selenium. The experiment used two media (one chondrogenic and one control) to culture hUCM-MSCs and TMJ condylar chondrocytes separately on PGA scaffolds before combining the seeded scaffolds to assess chondrogenic potential. Biochemical, histological, and immunohistochemical (IHC) analyses were subsequently conducted. Despite both the hUCM-MSC and TMJ condylar chondrocyte groups initially having an equal number of cells, a statistically significant increase in cell count was observed in the hUCM-MSC group after four weeks (4.75 × 10^6^, 3.06 × 10^6^ cells; respectively). Focusing on hUCM-MSCs, a significant increase was seen in both chondrogenic and control mediums at 4 weeks; notably, the cell count at this time point resulted in a higher cell count in the control medium compared to the chondrogenic medium (674 ± 173 × 10^3^, 638 ± 121 × 10^3^; respectively). Additionally, this biologic modality returned statistically significant increases in cell count compared to the condyle chondrocytes, regardless of medium. Histological analysis was conducted to evaluate the capacity of these therapies in producing a viable extracellular matrix (ECM) by quantifying glycosaminoglycan (GAG) content. The hUCM-MSCs did not reduce the GAG content as much as the condylar chondrocytes, but both interventions resulted in a decrease in GAG synthesis, irrespective of the medium used. At week 0, IHC analysis showed some type I and minimal type II collagen in the hUCM-MSC and TMJ constructs, supported by hydroxyproline assay (HPA) results. At week 4, IHC showed that the hUCM-MSC constructs showed similar type I and II collagen levels to baseline, but the evidence was not supported by HPA results, as the collagen levels were too low to be detected by the assay. At week 4, IHC staining was inconclusive for type I/II collagen in TMJ constructs in the control medium but showed minimal presence of both type I and II collagen in the chondrogenic medium (10).

Wang et al. researched the effects of human UC-MSCs for osteochondral tissue engineering purposes, using a sandwich approach. The stem cells were initially isolated and characterized via flow cytometry for certain cluster of differentiation (CD) surface markers including CD29, CD49e, CD73, CD90 and CD105. These hUC-MSCs were then seeded onto sterilized, poly-l-lactic acid (PLLA) scaffolds which were regularly enriched with either a chondrogenic or osteogenic medium. After 3 weeks, two PLLA scaffolds were sutured together to create 1 of 4 composite groups: 2 chondrogenic (C-C), 2 osteogenic (O-O), 1 chondrogenic and 1 osteogenic (C-O), or a layer of hUC-MSCs (10 *μ*l solution containing 100 × 10^6^/ml cells) placed between 1 chondrogenic and 1 osteogenic (C-cell-O) construct. After 6 weeks of culturing, the composites were separated; biochemical, immunohistochemical, histological, and reverse transcription polymerase chain reaction (RT-PCR) tests were conducted. The hUC-MSCs showed high levels of the aforementioned CD markers. Over a 6-week period, DNA content decreased in the chondrogenic groups, with a 40% decrease from weeks 0 to 3 (*p* < 0.05), while osteogenic constructs showed stable DNA content for the first 3 weeks before decreasing by 23% from weeks 3 to 6; the osteogenic groups consistently exhibited a greater DNA content than the chondrogenic groups at both 3 and 6 weeks. Regarding the chondrogenic groups, GAG content demonstrated statistically significant increases at 3 weeks compared to baseline, but their levels were maintained until 6 weeks. Similarly, the osteogenic group exhibited consistent GAG contents over the 6-week interval. Comparing both groups, the GAG content in the chondrogenic group was significantly higher than that of the osteogenic group at weeks 3 and 6. The C-C group showed statistically significant increases in hydroxyproline (HYP) content both from baseline to week 6, and when comparing the chondrogenic components of the C-O and C-cell-O groups. Similar to chondrogenic groups, the osteogenic groups showed greater HYP content over the 6-week period (*p* < 0.05), with no statistically significant differences between groups. When comparing both groups, the chondrogenic group had 4.6 and 4.9 times the amount of HYP at 3 and 6 weeks, respectively; furthermore, the chondrogenic parts of the C-O and C-cell-O had 3.1 and 3.5 times more HYP than their osteogenic counterparts, respectively. Over the 6 weeks, calcium content slightly increased from baseline in the chondrogenic group, but in the osteogenic group statistically significant increases were seen: the O-O scaffolds had 1.5 and 2.1 times the amount of calcium compared to the C-O and C-cell-O groups, respectively. Histology and IHC assessments were also conducted on the scaffolds. At week 3, the chondrogenic scaffold showed weak type 1 collagen and aggrecan, moderate GAG, and non-existent type II collagen and calcium staining; the osteogenic group demonstrated negative staining for type I collagen, aggrecan, and GAGs, but did positively stain for calcium and type II collagen. The chondrogenic groups exhibited increases in chondrogenic genes (i.e., type II collagen and aggrecan) at weeks 3 and 6 (*p* < 0.05), while type I collagen gene expression increased in all groups (*p* < 0.05); osteogenic groups showed varied gene expression changes over the 6-week culture period (11).

### 3.2. Pre-clinical studies

Umbilical cord MSCs may attenuate arthritic diseases by modulating immune responses and reducing inflammation (12, 13). More specifically, there are three preclinical studies that support the use of human umbilical cord components for the treatment of temporomandibular joint osteoarthritis (TMJ-OA).

Kim et al. aimed to show that hUCM-MSCs have potential cartilage-regenerating and anti-inflammatory effects in a rabbit model of monosodium iodoacetate-induced TMJ-OA. The authors randomly and equally divided 25 rabbits into 5 groups: no TMJ-OA control (*n* = 5), TMJ-OA induced without treatment (*n* = 5), TMJ-OA induced + DEX treatment (*n* = 5), TMJ-OA + hUCM-MSCs at 1 × 10^5^ cells/200 μl saline (*n* = 5), TMJ-OA induced + hUCM-MSCs at 5 × 10^5^ cells/200 μl saline (*n* = 5), and TMJ-OA induced + hUCM-MSCs at 1 × 10^6^ cells/200 μl saline (*n* = 5). The investigators induced TMJ-OA in select rabbit groups by injecting three mg of monosodium iodoacetic acid into their TMJs bilaterally. To investigate hUCM-MSCs’ protective and regenerative potential on TMJ cartilage, the authors compared the outcomes of cartilage regeneration between those rabbits which received injection treatments of different concentrations of hUCM-MSCs, dexamethasone only, and no treatments at all. hUCM-MSCs demonstrated anti-inflammatory effects at all tested concentrations comparable to that of dexamethasone injections. However, only the groups treated with hUCM-MSCs portrayed chondrogenic regeneration compared to other groups that received only dexamethasone or no treatment at all. The median hUCM-MSC dose (treated at the concentration of 5 × 10^5^ cells/200 µl saline) showed the most prominent cartilage protective effect and potential for cartilage regeneration. These effects were achieved through the upregulation of growth factors, extracellular matrix markers, and anti-inflammatory cytokines, as well as the downregulation of pro-inflammatory cytokines (14).

Sumarta et al. evaluated the regeneration of mandibular cartilage defects using hUC-MSCs implanted over a platelet-rich fibrin (PRF) scaffold. The study involved 20 male Wistar rats randomly divided into four experimental groups: a control group with untreated mandibular defects (C), a group with hUC-MSC implanted in mandibular defects (E1), a group with PRF implanted in mandibular defects (E2), and a group with hUC-MSC and PRF scaffold combined in mandibular defects (E3). After a period of 6 weeks, the authors observed significant regeneration of mandibular cartilage defects in the group where hUC-MSC and PRF scaffold were combined, as evidenced by the expression of various markers (i.e., Ki67, Sox9, FGF 18, type 2 collagen, and aggrecan) and histological evaluations. Although the groups implanted with either hUC-MSC or PRF alone did show evidence of increased expression of these markers, the combination of both hUC-MSC and PRF together showed a significantly higher level of expression, which suggests an additive effect when both biologics are used together (15).

Ward et al. investigated the anti-inflammatory effects of human umbilical cord perivascular mesenchymal cells (hUC-PVCS) and a cell lysate (CL) in CD1 mice models of TMJ inflammation. The authors divided the mice into three groups and performed TMJ injections of saline (control, *n* = 8), 1% CL (*n* = 8), or viable HUCPVCS (*n* = 8). For all groups, a systemic pro-inflammatory state was induced by hind-paw injections of carrageenan beforehand. TMJ samples treated with HUCPVCS or their CL derivatives demonstrated significantly lower concentrations of leukocytes in TMJ synovial tissue. The hind paws that were excised 48 h following injections with HUCPVCS or CL showed significantly decreased concentrations of inflammatory markers such as Tumor necrosis factor alpha (TNF-α) and myeloperoxidase (MPO) when compared to controls. Moreover, histologic examination of TMJ synovial tissue in the treated articular samples showed reduced signs of acute inflammation when compared to controls (16).

### 3.3. Clinical studies

Connelly et al. conducted a retrospective case series assessing postoperative outcomes of interpositional implantation of cryopreserved viable osteochondral allograft (CVOCA) combined with viable cryopreserved umbilical cord tissue (vCUT) allograft after TMJ discectomy in patients with internal derangement and/or degenerative joint disease (DJD). Inclusion criteria consisted of patients who presented with TMJ pain and dysfunction refractory to other treatments, diagnosis of disc displacement (DD) or DJD, CVOCA and vCUT implantation post-TMJ discectomy, along with documentation of preoperative/postoperative pain, functional outcomes, and quality of life (QoL) scores. Exclusion criteria included incomplete data sets for TMJ pain, function or QoL. The primary outcome measure was Visual Analogue Score (VAS) score, and secondary outcome measures were TMJ functionality [measured via Maximal Incisal Opening (MIO) score] and QoL [measured via Glasgow Benefit Inventory (GBI) score]. 9 patients (out of 12 enrolled) who met the inclusion/exclusion criteria were included in this study. The VAS score showed a significant reduction (*p* < 0.05) post-operatively (3.0 ± 3.0) compared to pre-operative score (9.0 ± 2.0). The MIO score increased post-operatively compared to pre-operative score, but the increase was not statistically significant. The average GBI score also showed a trend toward improvement for QoL. This study has several limitations including small sample size, lack of comparator group, short follow-up and retrospective nature. Despite these, it is one of the first clinical studies demonstrating safety of these allografts and potential efficacy for TMJ reconstruction. Stringent, prospective, multi-center, adequately powered, non-randomized and randomized controlled studies with longer follow-up are needed to validate this technique involving use of allografts for TMJ reconstruction.

### 3.4. Ongoing clinical studies

As of August 20, 2023 there is one trial registered on ClinicalTrials.gov involving the use of UC tissue and/or associated MSCs for the management of TMJ disorders and is summarized in Table 1. No trials were registered on the Chinese Clinical Trial Register (ChiCTR) or Clinical Trials Registry—India (CTRI) using the aforementioned search terms.

**Table 1:** Clinical trials registered on clinicalTrials.gov till August 20, 2023 involving the use of UC tissue and/or associated MSCs for the management of TMJ disorders.

Study identifier	Tissue/cell type	Study phase; estimated enrollment (*N*)	Primary outcome measure(s)	Recruitment status	Country
NCT05305833	Umbilical Cord derived mesenchymal stem cells and adipose tissue derived stromal vascular fraction	Phase I/II; *N* = 20	1) Change from Baseline Pain at 15th day [Time Frame: 15th day]	Recruiting	Turkey
			• Using by Visual Analogue Scale (VAS); higher scores mean worse outcome		
			2) Change from baseline maximum mouth opening at 15th day [Time Frame: 15th day]		
			• The distance between incisal edge of maxillary central incisor to the incisal edge of mandibular central incisor, when the mouth is opened as wide as possible painlessly		
			3) Change from baseline maximum mouth opening at 1 month [Time Frame: 1 month]		
			• The distance between incisal edge of maxillary central incisor to the incisal edge of mandibular central incisor, when the mouth is opened as wide as possible painlessly at first month; higher scores means a better outcome		
			4) Change from baseline maximum mouth opening at 3 months [Time Frame: 3 month]		
			• The distance between incisal edge of maxillary central incisor to the incisal edge of mandibular central incisor, when the mouth is opened as wide as possible painlessly at third month; higher scores means a better outcome		
			5) Change from baseline maximum mouth opening at 6 months [Time Frame: 6 month]		
			• The distance between incisal edge of maxillary central incisor to the incisal edge of mandibular central incisor, when the mouth is opened as wide as possible painlessly at 6th month, higher scores means a better outcome		
			6) Change from baseline pain at 1 month [Time Frame: 1 month]		
			• Using by Visual Analogue Scale (VAS)		
			7) Change from baseline pain at 3 month [Time Frame: 3 month]		
			• Using by Visual Analogue Scale (VAS)		
			8) Change from baseline pain at 6 month [Time Frame: 6 month]		
			• Using by Visual Analogue Scale (VAS) higher scores mean worse outcome		

## 4. Discussion

Only two basic science studies have been conducted investigating the effect of UC-MSCs on TMJ regeneration. Bailey et al. conducted a comparative study analyzing hUCM-MSCs and condylar chondrocytes for TMJ cartilage regeneration, and the results revealed that hUCM-MSCs exhibited significantly greater proliferative potential, highlighting their promising role in the field of regenerative medicine. Histologically, both interventions exhibited promising outcomes. However, the hUCM-MSCs did not decrease the GAG concentration as significantly as the condylar chondrocytes, suggesting that the immunotherapeutic potential of the MSCs might not be as pronounced as that of cells derived from the native tissue. In general, the chondrogenic medium did not have a significant impact on the synthesis of hUCM-MSCs, and these constructs showed greater cell proliferation than TMJ constructs, potentially from anti-apoptotic pathways or increased adhesion in hUCM-MSCs. The decreased collagen synthesis at week 4 was unexpected, as the IHC staining showed collagen presence, indicating a denser collagen matrix (10). Wang et al.'s basic science investigation determined that hUC-MSCs were capable of differentiating towards either chondrogenicity or osteogenicity based on the received signals at 3 weeks. The osteogenic group was especially interesting, as RUNX2 gene expression was significantly upregulated at 3 weeks, but the levels of other osteogenic genes (i.e., OSX, BSP, and OCN) did not reach levels in par with mature osteoblastic states until 6 weeks, indicating that the differentiation media may need to be adjusted in future experiments. Between weeks 3 to 6, the chondrogenic component of any of the three scaffolds did not appear to undergo any osteogenic differentiation, and similarly none of the osteogenic components underwent any chondrogenic differentiation during the same time interval; this may indicate that the differentiation characteristics of the cell must have been confirmed within the first three weeks of culturing. Another significant finding from this study was the identification of improved integration when an intermediate layer of undifferentiated cells was placed between the osteogenic and chondrogenic components (11). Both Bailey et al. and Wang et al. delved into the potential of hUCM-MSCs for cartilage regeneration. Bailey et al. highlighted the superior cell proliferation of hUCM-MSCs compared to TMJ condylar chondrocytes, especially in the control medium. This suggests a robust inherent growth potential of hUCM-MSCs. On the other hand, Wang et al. emphasized the versatility of hUCM-MSCs in osteochondral tissue engineering, with the cells showing high levels of CD markers and significant changes in DNA and GAG content. The sandwich approach used by Wang et al. further underscores the adaptability of these cells in different tissue engineering contexts.

Three preclinical studies were incorporated into the present study. Kim et al. investigated the therapeutic effects of UC-MSCs on rabbit models of TMJ-OA. This investigation showed that even a single, intraarticular injection of UC-MSCs results in therapeutic effects, attributed to upregulation in expression of growth factors, ECM markers and anti-inflammatory cytokines and downregulation in expression of pro-inflammatory cytokines. This is clinically relevant, as such treatment reduces the financial burden on patients and healthcare system while obviating any pain or discomfort associated with repeated injections. Although the median hUCM-MSC dose (concentration of 5 × 10^5^ cells/200 µl saline) showed the most prominent cartilage protective effect and potential for cartilage regeneration, different severities of osteoarthritis may need higher doses or repeat injections. However, these findings allow for pre-clinical estimates of hUCM-MSC dosing efficacy prior to clinical trial implementations, expediting their safe in-vivo use. UC-MSCs are a viable therapy for TMJ-OA, as their therapeutic potential manifests with chondrogenic regenerative effects (14). Sumarta et al. studied the effect of hUC-MSCs with a PRF scaffold on mandibular cartilage defects in CD1 rat models. The stem cells were seeded onto the PRF scaffold and subsequently cultured in a chondrogenic medium, leading to ECM deposition. The E3 group showed significantly more chondrogenic regenerative potential, most likely from the addition of the PRF scaffold (i.e., increased strength, more growth factors, greater healing/integration potential). The proliferative capacity of hUC-MSCs in the PRF scaffold (indicated by strong Ki67 expression) was potentially influenced by PRF's ECM, which facilitates cell signaling, diffusion of growth factors, and mechanical signal reduction, potentially involving platelet-derived growth factor-mediated Akt signaling; these findings align with the observed elevated chondrocyte counts. High chondrocyte counts correspond to significant FGF18 expression, as seen in the E3 group compared to the other groups, suggesting potential chondrogenic differentiation through FGFR3-mediated pathways, further enhancing type 2 collagen production; moreover, SOX9's induction of chondrogenesis via Smad2/3 was observed after six weeks of hUC-MSC implantation in a PRF scaffold, potentially influenced by FGFR3, as implied by the high concurrent expression of other related genes (i.e., Aggrecan, FGF18, Sox9, and type 2 collagen). The regenerative capability of hUC-MSCs within a PRF scaffold was assessed measuring the average cartilage thickness. Overall, this study showed the promising potential of hUC-MSCs on mandibular cartilage defects (15). As the last preclinical study that fit the scope of our article, Ward et al.'s study demonstrates that hUC-PVSCs can suppress the infiltration of inflammatory markers/leukocytes and decrease the amount of edematous tissue that occurs as a reaction to injury (16). The three preclinical studies by Kim et al., Sumarta et al., and Ward et al., provide a glimpse into the *in vivo* potential of umbilical cord MSCs. Kim et al.'s study on rabbits showcased both the anti-inflammatory and cartilage-regenerating effects of hUCM-MSCs, with the medium dose showing the most pronounced effects. This dose-dependent response is crucial for future therapeutic considerations. Sumatra et al., on the other hand, highlighted the synergistic effects of combining hUC-MSCs with a PRF scaffold, suggesting that the therapeutic efficacy of MSCs can be enhanced with the right scaffold. Ward et al.'s focus on the anti-inflammatory effects of hUC-PVCS and their cell lysate in mouse models further supports the anti-inflammatory potential observed by Kim et al.

Based on our search strategy and inclusion criteria, only one clinical study fits the scope of our article. Connelly et al. aimed to assess the outcomes of implantation of CVOCA and vCUT allografts after TMJ discectomy for patients with degenerative diseases of the TMJ, hypothesizing functional TMJ restoration with limited donor site morbidity, and using post-operative pain and MIO/GBI scores as endpoints. CVOCA and vCUT allografts were selected as the intervention of choice based on previously published, positive outcomes (i.e., tendon repair, lack of adverse events, effective wound healing) in reconstructive procedures. Twelve patients received CVOCA and vCUT implants after a TMJ discectomy, with promising results showing pain reduction, increased ability to open their mouth, and improved quality of life scores. Positive trends were observed in both MIO and GBI scores, but statistical significance was not achieved for MIO, a consequence of the small sample size or short follow-up time. Overall, this study's clinical outcomes aligned with previous data, but instead of relying on autologous tissue replacement, the use of allograft implants helped to eliminate donor site morbidity, shorten surgery duration, and potentially promote tissue regeneration, with no graft failures, complications, or additional interventions recorded during follow-up (17). Our review of the current literature only yielded one clinical study that fits the scope of our inclusion and exclusion criteria, which limits our ability to critically compare different findings regarding UC components accordingly. The study by Connelly et al. assessed the postoperative outcomes in patients receiving different allografts. The authors showed promising results with both CVOCA and vCUT allografts, but further studies involving direct comparison between the two or against a non-allograft control are necessary to reach more definitive conclusions on therapeutic equivalence or superiority.

## 5. Conclusion

This review acts as a compilation of basic science, preclinical, and clinical investigations on the therapeutic use of UC tissue and derived MSCs for the treatment of injuries to the temporomandibular joint. This article illustrates the strong evidence supporting the safety and effectiveness of biologics derived from the UC. Further non-randomized or randomized, *in vitro*, pre-clinical, and clinical trials with multi-year follow-up time intervals are necessary to endorse the clinical use of this intervention.
